# Imaging microvascular changes in nonocular oncological clinical applications by optical coherence tomography angiography: a literature review

**DOI:** 10.2478/raon-2023-0057

**Published:** 2023-11-30

**Authors:** Rok Hren, Gregor Sersa, Urban Simoncic, Matija Milanic

**Affiliations:** Faculty of Mathematics and Physics, Ljubljana, Slovenia; Institute of Mathematics, Physics, and Mechanics, Ljubljana, Slovenia; Syreon Research Institute, Budapest, Hungary; Institute of Oncology Ljubljana, Ljubljana, Slovenia; Jozef Stefan Institute, Ljubljana, Slovenia

**Keywords:** optical coherence tomography angiography (OCTA), oncology, endoscopy, skin carcinoma

## Abstract

**Background:**

Optical coherence tomography angiography (OCTA) is an emerging imaging modality that enables noninvasive visualization and analysis of tumor vasculature. OCTA has been particularly useful in clinical ocular oncology, while in this article, we evaluated OCTA in assessing microvascular changes in clinical nonocular oncology through a systematic review of the literature.

**Method:**

The inclusion criterion for the literature search in PubMed, Web of Science and Scopus electronic databases was the use of OCTA in nonocular clinical oncology, meaning that all ocular clinical studies and all ocular and nonocular animal, phantom, ex vivo, experimental, research and development, and purely methodological studies were excluded.

**Results:**

Eleven articles met the inclusion criteria. The anatomic locations of the neoplasms in the selected articles were the gastrointestinal tract (2 articles), head and neck (1 article) and skin (8 articles).

**Conclusions:**

While OCTA has shown great advancements in ophthalmology, its translation to the nonocular clinical oncology setting presents several limitations, with a lack of standardized protocols and interpretation guidelines posing the most significant challenge.

## Introduction

It was demonstrated that angiogenesis is closely associated with tumor growth, as the development of vasculature has the capacity to supply oxygen and nutrients to dividing tumor cells.^1^ Microvascular alterations are therefore typical signatures of early tumor development and progression. Conventional techniques for assessing microvascular changes are narrow band imaging (NBI)^23^ and confocal laser endomicroscopy (CLE)^45^ in endoscopy and confocal laser microscopy (CLM)^6^ and dermoscopy^7^ in skin diagnosis. However, NBI has limited resolution, and CLE utilizes exogenous tracers, while CLM and dermoscopy cannot visualize deeper vascular changes due to a limited penetration depth, with blood vessels also often being hidden in pigmented lesions. To address these shortcomings, various emerging imaging techniques have been explored for microvascular imaging.

**FIGURE 1.**
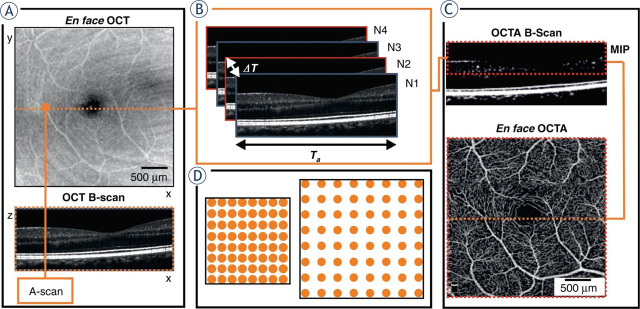
Optical coherence angiography (OCTA) scanning protocol. **(A)** A raster scanning protocol for blood vessel visualization, with the x-axis sampling density determined by A-scans per B-scan sets and the y-axis determined by B scans per volume sets. **(B)** OCTA B-scans created by four repeated B-scans at one y-location, repeated for varying positions along the y-axis, impacting sampling density; ΔT represents interscan time and T_a_ denotes acquisition time. **(C)** Maximum intensity projection (MIP) applied to the OCTA B-scan within the depth range of interest (where vessels are located) to generate one line of the en face OCTA image. **(D)** Illustration depicting the equal distribution of sampling points for smaller and larger imaging areas. Taken from Sampson *et al.*^12^ and reprinted with permission from the publisher by the Creative Commons license. To view a copy of Creative Commons license, visit http://creativecommons.org/licenses/by/4.0/.

Optical coherence tomography (OCT) is a mature imaging technique that uses low-coherence light to capture high-resolution, cross-sectional images of biological tissues in real time. It has been applied in various fields of medicine^8^ due to its noninvasiveness, high resolution, and ability to visualize microstructure and has become the gold standard for diagnosis in ophthalmology.^9^ It is thus not surprising that OCT has found its way into oncological applications as well.^10^ To enable further functional assessment of tumors, optical coherence tomography angiography (OCTA) is an impending valuable extension of OCT in oncological research and clinical practice. OCTA is a modification of OCT and works by comparing the light waves that are reflected from stationary tissue with the light waves that are reflected from moving red blood cells (RBCs), and this information is then used to create a detailed map of the blood vessels (Figure 1). The distinct advantage of OCTA is that it is a noncontact, nonionizing, and noninvasive modality and does not require a contrast agent. OCTA has proven highly valuable in helping to better understand and manage a range of nononcological ocular pathologies^11,12,13^, while in oncological ocular clinical applications, OCTA has potential for use in the diagnosis and monitoring of chorioretinal pathologies, such as neovascularization and macular edema.^14^

How valuable OCTA could be in quantifying microvascular changes in nonocular clinical oncology remains unclear, and to that end, we decided to systematically review the literature with the intention of exclusively focusing only on studies in which OCTA was performed on patients in the clinical oncology setting.

## Materials and methods

Two authors (R.H. and M.M.) conducted jointly—to preclude potential bias—a comprehensive literature search on August 3, 2023, through PubMed, Web of Science and Scopus electronic databases using the following search terms: “optical coherence tomography angiography tumors” and “dynamic optical coherence tomography tumors”. No restrictions on publication date or language were imposed. The inclusion criterion was the nonocular application of OCTA in the oncological clinical setting, meaning that all ocular oncological clinical studies and all ocular and nonocular animal and phantom, *ex vivo*, experimental, research and development, and purely methodological studies were excluded. Special care was taken that duplications were removed, both across databases and across studies; for example, if the study was first published in proceedings and later in the journal, then proceedings article was considered a nonprimary publication and therefore excluded. Studies were categorized with respect to the anatomical location of the tumors.

**TABLE 1.** Included articles reporting the use of optical coherence tomography angiography (OCTA) to quantify microvascular changes in nonocular clinical applications in oncology

**Reference**	**Year of publication**	**Number of patients**	**Oncologic setting**
***GI tract***			
**Tsai *et al.*^15^**	2014	1	Nondysplastic Barrett's esophagus
**Lee *et al.*^16^**	2017	52	Nondysplastic Barrett's esophagus surveillance or endoscopic eradication therapies for low-grade/high-grade dysplasia
***Head and neck***			
**Maslennikova *et al.*^17^**	2017	25	Radiotherapy of oropharyngeal and nasopharyngeal cancer
***Skin***			
**De Carvalho *et al.*^18^**	2016	1	Naevus to melanoma transition
**Themstrup *et al.*^19^**	2017	47	Actinic keratosis, Bowen's disease and squamous cell carcinoma
**Themstrup *et al.*^20^**	2018	81	Basal cell carcinoma
**Meiburger *et al.*^21^**	2019	7	Basal cell carcinoma
**Gubarkova *et al.*^22^**	2019	27	Basal cell carcinoma
**De Carvalho *et al.*^23^**	2018	127	Melanoma
**Welzel *et al.*^24^**	2021	159	Melanoma
**Perwein *et al.*^25^**	2023	130	Nevi

GI = gastrointestinal

## Results

In total, 3977 articles were found to be of interest in the PubMed, Web of Science and Scopus databases; it is noteworthy that 3855 articles (96.9% of total) were linked to ocular oncological studies. After excluding duplicates and applying the exclusion criteria, first considering the title and abstract and then, if necessary, reading the entire article, 11 articles were identified for further analysis. The anatomical locations of tumors in the selected articles were the gastrointestinal (GI) tract (2 articles), head and neck (1 article) and skin (8 articles).

### GI tract

**FIGURE 2.**
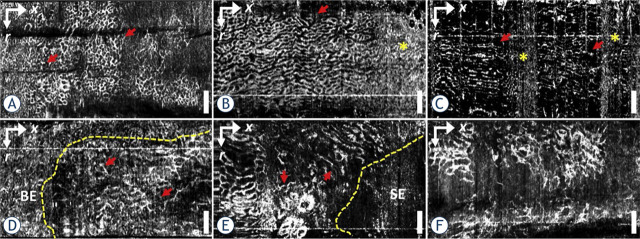
Images obtained through optical coherence tomography angiography (OCTA) for **(TOP ROW)** nondysplastic Barrett's esophagus (NDBE) and **(BOTTOM ROW)** low-grade/high-grade dysplasia (LGD/HGD) (bottom left LGD; bottom center and bottom right HGD). NDBE images show a regular honeycomb microvascular pattern (arrows, top row), while abnormal vascular features, such as abnormal vessel branching (arrows, bottom left), heterogeneous vessel size (arrows, bottom center) or both (bottom right), are observed in LGD/HGD. Motion artifacts are denoted by asterisks. OCTA images can assist in distinguishing the boundary between abnormal microvasculature and neighboring nondysplastic regions (dashed line, bottom left and bottom center). Taken from Lee *et al*.^16^ and reprinted with permission from the publisher.

A pioneering effort in assessing microvasculature by means of OCTA in clinical oncology was the work of Tsai *et al.*^15^ They applied a modality to image subsurface vascular patterns in a patient with nondysplastic Barrett's esophagus (NDBE) and demonstrated that in this way, the diagnostic capability of endoscopic OCT was enhanced. Lee *et al.*^16^ continued their work and collected 97 datasets from 52 patients with NDBE and low-grade/high-grade dysplasia (LGD/HGD). Their goal was to differentiate NDBE patients from LGD/HGD patients; however, due to insufficient image quality, 43 datasets (44%) in 20 patients were not used for analysis; OCTA images were also not generated in real time due to the high computational burden. The findings of the study revealed distinct differences in microvascular OCTA features of abnormal vessel branching and heterogeneous vessel size between the NDBE and LGD/HGD groups, as shown in Figure 2. Further research with a larger patient population is required to validate these findings and establish the clinical utility of endoscopic OCTA in routine practice.

### Head and neck

**FIGURE 3.**
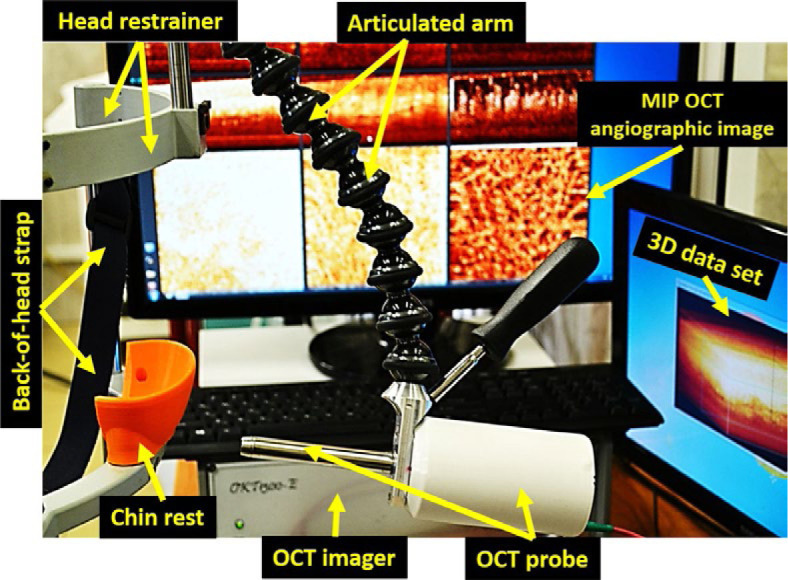
Optical coherence tomography angiography (OCTA) acquisition system. OCTA images were acquired in real time. Taken from Maslennikova *et al.*^17^ and reprinted with permission from the publisher by the Creative Commons license. To view a copy of Creative Commons license, visit http://creativecommons.org/licenses/by/4.0/.

In the study by Maslennikova *et al.*^17^, clinicians aimed to investigate the use of OCTA for imaging microvascular changes in the oral mucosa of cancer patients undergoing radiotherapy (RT). Authors conducted a longitudinal study involving 25 patients with oropharyngeal and nasopharyngeal cancer undergoing RT. OCTA was employed to visualize and analyze the microvascular network within the oral mucosa over time, and imaging was performed before the treatment and at regular intervals during and after RT. OCTA images were generated in real time by the acquisition system shown in Figure 3. The findings of the study demonstrated significant alterations in the microvascular morphology and density in the irradiated oral mucosa over the course of RT, with the microvascular network showing an increase in the vascular density and total length of capillary-like vessels compared to the baseline measurements. These changes were found to be more prominent when grade two and three mucositis developed. The study demonstrated the potential of OCTA as a valuable tool for longitudinal monitoring of microvascular changes in radiation-induced oral mucosal damage. However, it is important to note that this study has several limitations, as the sample size was relatively small, and the results may not be generalizable to the broader population; additionally, the prognostic significance of the observed microvascular changes needs further investigation.

### Skin

**FIGURE 4.**
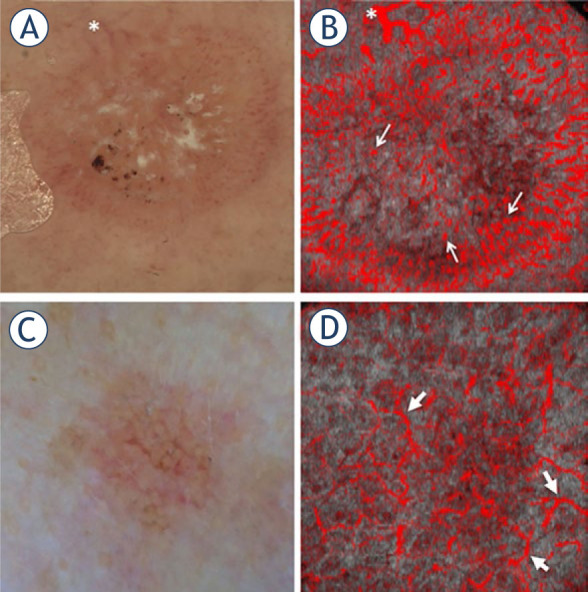
Illustration of two distinct vascular features observed through dermoscopy and optical coherence tomography angiography (OCTA). The first feature, referred to as “blobs”, is small, isolated points with a simple round appearance; the second feature, called “curves”, is narrow, curved, continuous structures of varying length. Panel **(A)** displays a dermoscopic image of a Bowen's disease (BD) lesion, and panel **(B)** shows the corresponding OCTA image. The asterisk in both panels points to the same vessel. The thin arrows in panel **(B)** indicate examples of vascular blobs. Similarly, panels **(C)** and **(D)** display a dermoscopic image of AK and the corresponding OCTA image, respectively. The thick arrows in panel **(D)** indicate examples of vascular curves. Taken from Themstrup *et al.*^19^ and reprinted with permission from the publisher.

De Carvalho *et al.*^18^ published a case report in which they showed an increased vasculature in the melanoma region compared to the nevus. Following this, Themstrup *et al.*^19^ conducted a study to distinguish subtypes within the keratinocyte skin cancer spectrum enrolling 18 patients with actinic keratosis (AK), 12 patients with Bowen's disease (BD) and 24 patients with squamous cell carcinoma (SCC). In this exploratory clinical study, they identified two vascular features that showed significant differences across the lesion types. One of these vascular features, referred to as “blobs”, i.e., small, isolated points with a simple round appearance, was more frequently present in BD cases but either absent or only slightly present in AK and SCC lesions. The other feature, called “curves”, i.e., narrow, curved, continuous structures of varying length, was predominantly present in AK lesions. These findings are illustrated in Figure 4.

In a subsequent study^20^, the same group continued with the differentiation of common basal cell carcinoma (BCC) subtypes by scanning 81 patients with 98 BCC lesions, of which 27 were superficial BCC (sBCC), 55 were nodular BCC (nBCC) and 16 were infiltrative BCC (iBCC). In this study, they found various structural and microvascular features that would aid in identifying nBCC, iBCC and sBCC subtypes. For example, it was shown that the presence of so-called “serpiginous” vessels, i.e., wavy structures of varying length, indicated an increased risk of nBCC and a reduced risk of sBCC.

Meiburger *et al.*^21^ applied OCTA to a patient with nBCC and six patients with sBCC and developed an algorithm for automatically determining skin lesion area using vascular density. While authors were hopeful in their conclusion that proposed method could facilitate diagnosis and treatment of BCC, no further study was published.

Gubarkova *et al.*^22^ examined 27 patients with BCC who received photodynamic therapy (PDT). They utilized OCTA imaging before and immediately after PDT and during follow-up visits to monitor vascular changes. Analysis of the OCTA images allowed for quantification of parameters such as blood vessel density and uniformity, aiding in distinguishing among BCC subtypes. The study demonstrated that OCTA offers real-time information on vascular changes in response to PDT. The researchers observed a decrease in blood vessel density at 24 hours after PDT, with OCTA images having 97% predictive value for differentiation between complete and partial responders.

**FIGURE 5.**
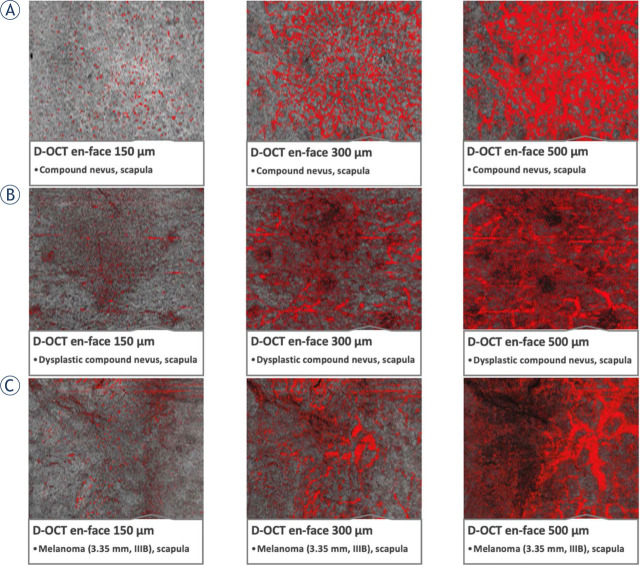
Microvascularization in skin lesions (nevi, dysplastic nevi, and melanomas) through optical coherence tomography angiography (OCTA) scans (denoted as D-OCT). **(A)** Compound nevus on the scapula, displaying a globular appearance with recent changes. **(B)** Dysplastic nevus, flat lesion on the scapula, exhibiting a complex appearance, atypical network, irregular pigmentation, and dots/globules. **(C)** Melanoma, a lesion on the scapula, measuring 3.35 mm in tumor thickness, classified as pT3aN1bM0S2, stage IIIB. Taken from Perwein *et al.*^25^ and reprinted with permission from the publisher by the Creative Commons license. To view a copy of Creative Commons license, visit http://creativecommons.org/licenses/by/4.0/.

De Carvalho *et al.*^23^ conducted a systematic analysis of melanoma lesions in 127 patients and found a significant link between specific microvascular features and Breslow's thickness. In a more recent study, Welzel *et al.*^24^ assessed 159 melanomas from 156 consecutive patients and found that irregular vascular shapes, including blobs, curves and serpiginious vessels, were more common in high-risk and metastatic melanomas than in low-risk lesions. Most recently, the same group^25^ prospectively examined a total of 167 nevi, including dysplastic ones, in 130 participants and compared these microvascular features to those found earlier in 159 melanomas.^24^ They found that increased blood vessel density and diameter and irregular tissue architecture were associated with melanomas, while nevi showed more regular structures and lower blood vessel density and diameter, indicating their benign nature (Figure 5). Researchers also found excellent predictive diagnostic value of microvascular features (e.g., blobs, serpiginious vessels) for nevi (88.2% to 91%) and melanoma (95.5% to 96.8%) and concluded that OCTA “may be a valuable addition to the current clinical-dermoscopic gold standard”.^25^

## Discussion

Based on this literature review, the inference could be made that OCTA is still finding its place in oncological clinical applications. It appears that the translation of OCTA from ocular applications to the nonocular clinical oncology setting faces certain limitations that could potentially hinder its widespread adoption.

### Limited penetration depth

One of the obvious limitations of OCTA in nonocular clinical oncology settings is its restricted penetration depth. OCTA relies on detecting motion contrast generated by moving RBCs, which limits its applicability to superficial structures. Tumors and lesions in deeper anatomical locations, such as within organs or soft tissues, may not be adequately visualized using OCTA due to limited tissue penetration. This constraint hampers its potential for comprehensive evaluation and monitoring of oncological conditions.

However, this limitation can be overcome by using endoscopic techniques bringing the instrument closer to the tissue of interest. As demonstrated in the GI tract studies by Tsai *et al.*^15^ and Lee *et al*.^16^, OCTA can be used endoscopically. Namely, common OCT has been developed as endoscopic probes of different types and used to obtain microscopy images of entire luminal organs, solid tumors, or vessels.^26^ Since OCTA is an extension of OCT, the same already developed technology can be used to bring the system closer to the tissue of interest.

Another possibility to increase the OCTA penetration depth is to use OCTA systems with longer wavelengths. In the studies presented in this article, the OCTA systems utilized 1.3 μm wavelengths, which is a typical wavelength also used for skin imaging; in ophthalmology, a shorter wavelength of 0.8 μm is typically used, resulting in an approximately 60% lower penetration depth. In a recent publication by Nishizawa and Yamanaka^27^, it was shown that by using a 1.7 μm wavelength, the penetration depth increases by approximately 40% compared to a 1.3 μm wavelength. Therefore, by developing OCTA systems with even longer wavelengths, larger penetration depths could be obtained.

### Inability to differentiate vessel types

OCTA provides detailed structural information about blood vessels but lacks the ability to differentiate between different vessel types. In the field of oncology, the distinction between arterial and venous vasculatures is crucial, as tumor angiogenesis is primarily associated with the growth of new abnormal blood vessels. Accurate differentiation between these types of vessels aids in assessing tumor progression and treatment response. Unfortunately, OCTA's current capabilities fail to provide this level of vessel characterization, limiting its effectiveness in nonocular oncological settings.

In ophthalmology, recent articles report the possibility of differentiating between arteries and veins utilizing various OCTA image parameters, including vascular diameters and shape and perfusion intensity density.^28^ However, the current methods for artery-vein classification in OCTA employ complex algorithms, thereby making it difficult for clinical applications. To alleviate this hindrance, deep learning algorithms were developed to reduce the complexity and automate artery-vein classification.^29^ Similar algorithms should also be developed for other OCTA modalities.

### Motion artifacts

Movement, including patient motion during OCTA acquisition, can introduce motion artifacts, leading to image distortions and reduced image quality. Unlike ophthalmology, where patients can fixate on a target, patients in nonocular oncology settings often have limited control over motion, making motion artifacts more challenging to mitigate. This limitation can compromise the accuracy and reliability of OCTA in nonocular clinical oncology, demanding the need for advanced postprocessing algorithms to improve image quality.

Since motion artifacts are well-known sources of artifacts in OCT imaging, they have been extensively researched. One possibility is to detect and compensate for the axial motion artifacts pixelwise by comparing the topology of different layers in tissue, and the motion artifacts are then compensated by shifting the pixel numbers with the value detected.^29^ Another possibility is to remove the affected scans in the software and to use only the nonaffected scans for vasculature image reconstruction.^30^ However, this approach may increase the duration of imaging sessions; therefore, it would be better to use an approach without the need for rescanning. As a solution, it was demonstrated that the motion contribution to the OCT signal can be reasonably estimated by considering statistics of the measured flow signal across all voxels.^30^ By implementing motion artifact compensation strategies, the translation of OCTA to clinical workflow would become more feasible.

### Lack of standardized protocols and interpretation

The lack of standardized protocols and interpretation guidelines is a significant limitation of OCTA in nonocular clinical oncology. Unlike ophthalmology, where standardized imaging protocols and interpretation criteria exist, the application of OCTA in oncology lacks such standardization. As a result, different centers may use varying acquisition settings, image processing algorithms, or interpretation approaches, leading to inconsistent and noncomparable results. Establishing standardized protocols and guidelines specific to nonocular oncology would enhance the accuracy and reproducibility of OCTA findings.

While OCTA has shown great promise in ophthalmology, its translation to the nonocular clinical oncology setting faces limitations. In particular, the lack of standardized protocols and interpretation guidelines poses a significant challenge. Addressing these limitations through advancements in technology, algorithm development, and a larger number of clinical sites initiating clinical trials is essential for realizing the full potential of OCTA in nonocular clinical oncology.

## Supplementary Material

Supplementary Material Details
