# Supplementary material for: Imaging microvascular changes in nonocular oncological clinical applications by optical coherence tomography angiography: a literature review
Source: Radiol Oncol. 2023 Nov 30;57(4):411–8. doi: 10.2478/raon-2023-0057 (PMC10690745; doi:10.2478/raon-2023-0057)
Supplement: Supplementary file 1 — Supplementary Material Details [file raon-2023-0057_sm.pdf]

Locoregional therapy combined with systemic therapy (LRT + ST) for unresectable and metastatic intrahepatic cholangiocarcinoma: a systematic review and meta-analysis

Mengqi Zhang, Weiwei Qi, Xiaofei Qiu, Chunpeng Yu, Wensheng Qiu, Song Wang, Zhenkang Qiu

doi: 10.2478/raon-2023-0059

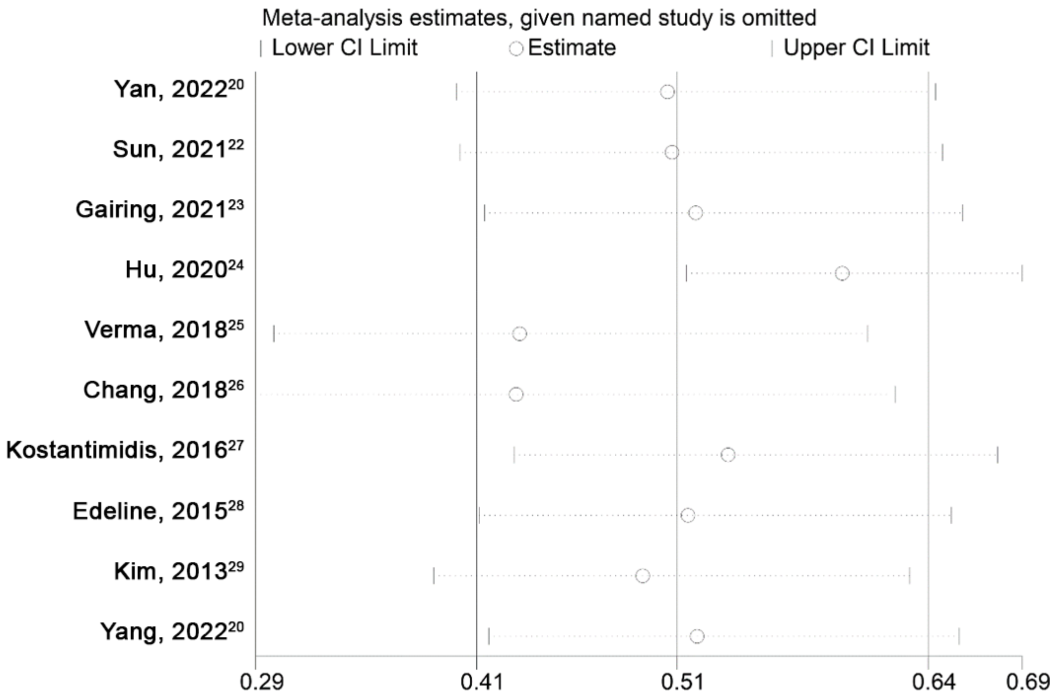

SUPPLEMENTARY FIGURE 1. Sensitivity analysis of overall survival (OS) in unresectable intrahepatic cholangiocarcinoma (iCCA) patients from the ten included articles.

CI = Confidence intervals

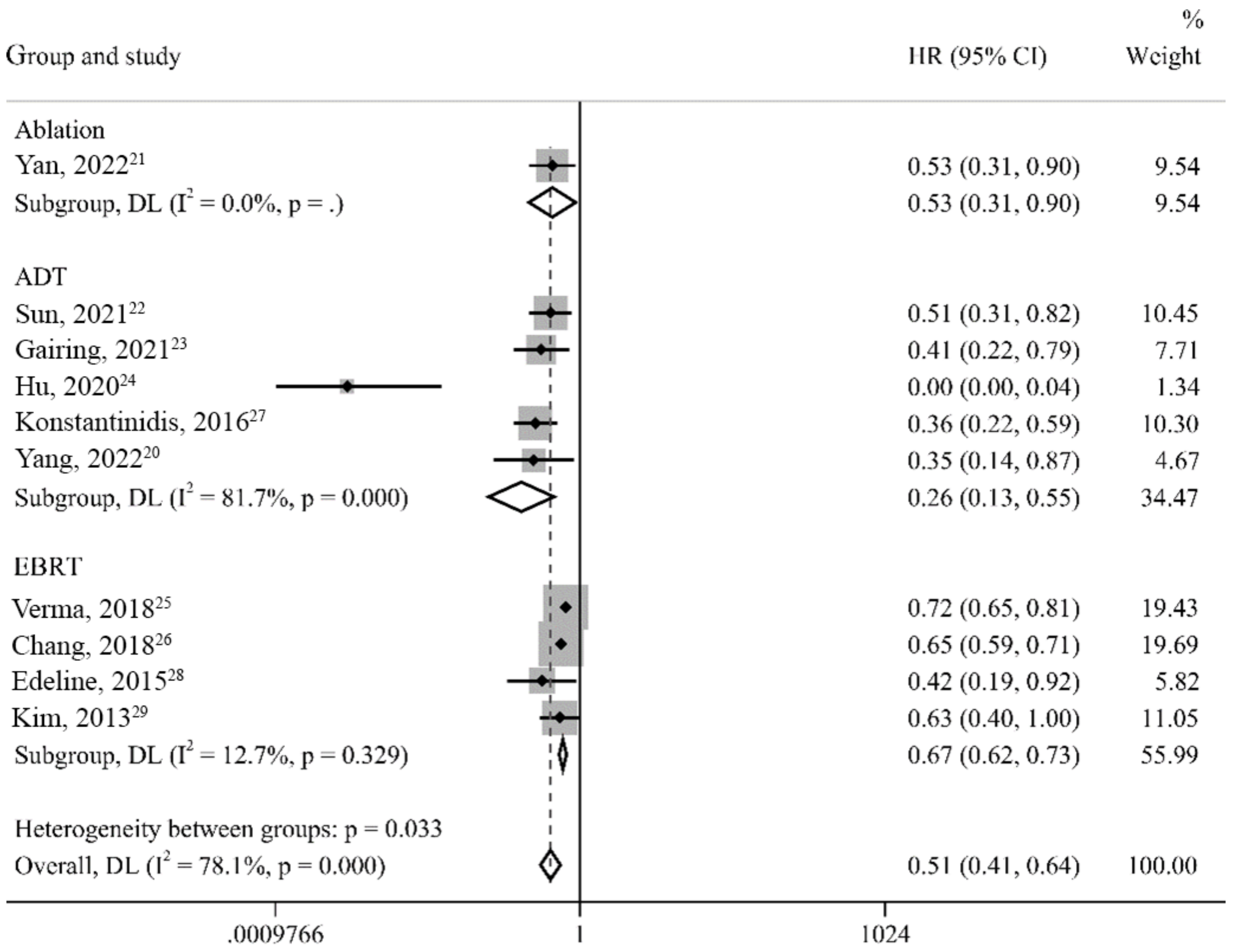

SUPPLEMENTARY FIGURE 2. Subgroup analysis of overall survival (OS) in unresectable intrahepatic cholangiocarcinoma (iCCA) patients from all ten included studies according to types of locoregional plus systemic therapy (ablation, ADT, EBRT).

Weights and between-subgroup heterogeneity test are from random-effects model

95% CI = 95% confidence intervals; ADT = Arterially directed therapy; DL = DerSimonian–Laird method; EBRT = External beam radiation therapy; HR = Hazard ratio; IV = Inverse variance method

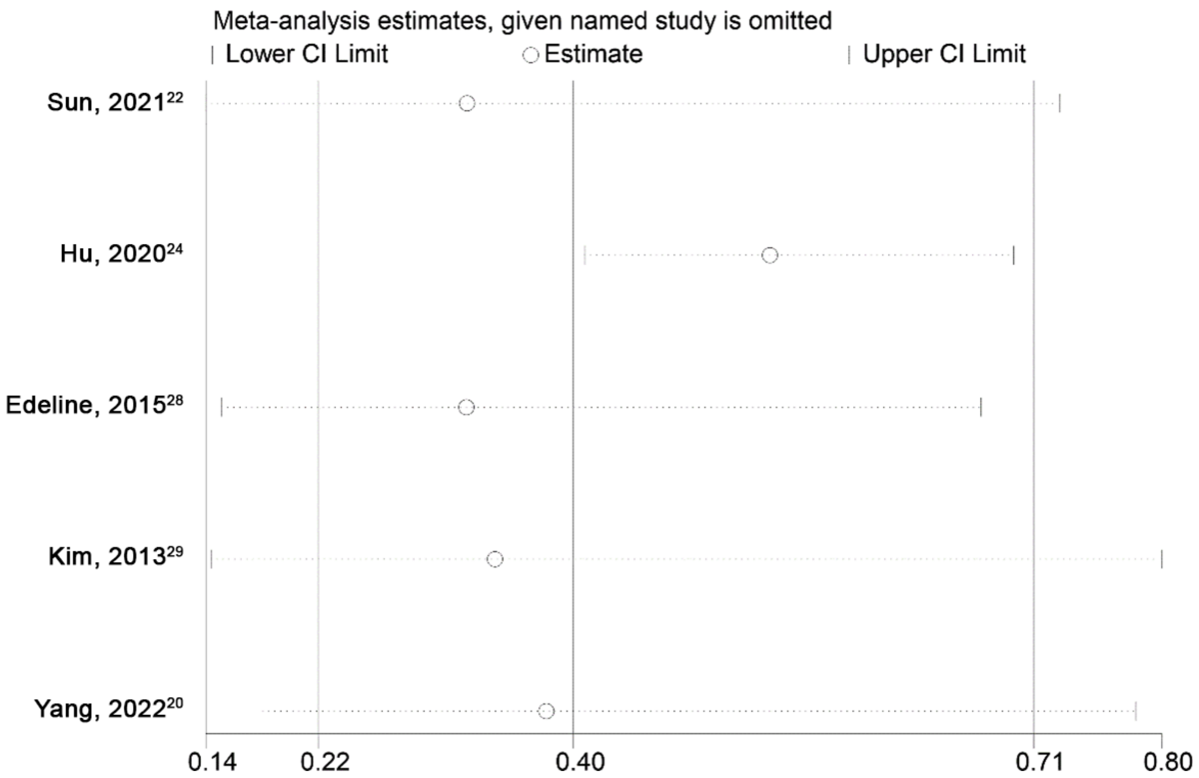

SUPPLEMENTARY FIGURE 3. Sensitivity analysis of progression-free survival (PFS) in unresectable intrahepatic cholangiocarcinoma (iCCA) patients from five included articles.

CI = Confidence intervals

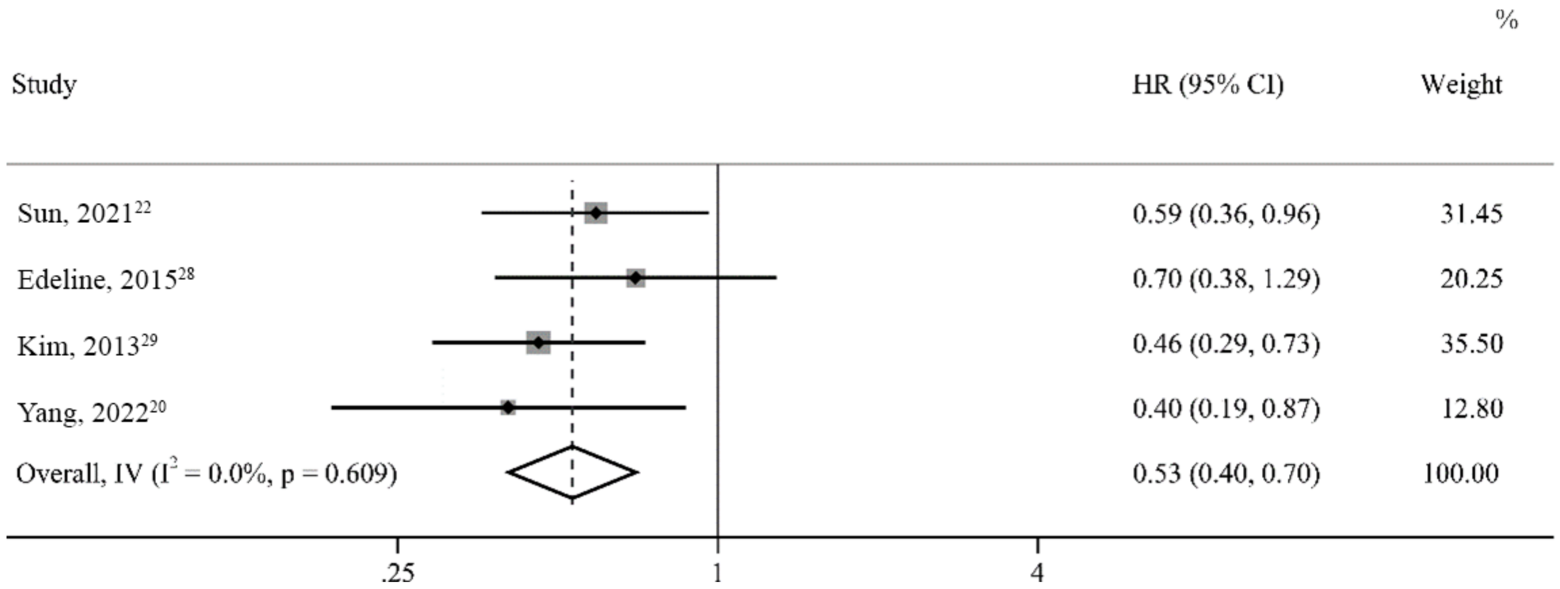

SUPPLEMENTARY FIGURE 4. Forest plots for progression-free survival (PFS) in unresectable intrahepatic cholangiocarcinoma (iCCA) patients from four studies except for Hu's study.

95% CI = 95% confidence intervals; HR = Hazard ratio; IV = Inverse variance method

SUPPLEMENTARY TABLE 1. Detailed search queries

Database	Query
PubMed	("logical"[Title/Abstract] OR "Locoregional"[Title/Abstract] OR "LRT"[Title/Abstract] OR "embolization"[Title/Abstract] OR "Embolization, Therapeutic"[Mesh] OR "Embolization, Therapeutic"[Title/Abstract] OR "Chemoembolization, Therapeutic"[Mesh] OR "Chemoembolization, Therapeutic"[Title/Abstract] OR "chemoembolization"[Title/Abstract] OR "radioembolization"[Title/Abstract] OR "TACE"[Title/Abstract] OR "TARE"[Title/Abstract] OR "hepatic arterial infusion"[Title/Abstract] OR "HAI"[Title/Abstract] OR "intra-arterial therapy"[Title/Abstract] OR "Ablation Techniques"[Mesh] OR "Ablation Techniques"[Title/Abstract] OR "Ablation"[Title/Abstract] OR "MWA"[Title/Abstract] OR "RFA"[Title/Abstract] OR "Cryoablation"[Title/Abstract] OR "Y-90"[Title/Abstract] OR "Radiotherapy"[Mesh] OR "Radiotherapy"[Title/Abstract] OR "radiation"[Title/Abstract] OR "Chemoradiotherapy"[Mesh] OR "Chemoradiotherapy"[Title/Abstract] OR "EBRT"[Title/Abstract] OR "SBRT"[Title/Abstract] OR "Chemotherapy"[Title/Abstract] OR "systemic treatment"[Title/Abstract] OR "Systemic Therapy"[Title/Abstract] OR "Sorafenib"[Title/Abstract] OR "Lenvatinib"[Title/Abstract] OR "Atezolizumab and bevacizumab"[Title/Abstract] OR "regorafenib"[Title/Abstract] OR "Cabozantinib"[Title/Abstract] OR "ramucirumab"[Title/Abstract] OR "Nivolumab"[Title/Abstract] OR "Pembrolizumab"[Title/Abstract] OR "PD-1"[Title/Abstract] OR "PD-L1"[Title/Abstract] OR "Combined Modality Therapy"[Mesh] OR "Combined Modality Therapy"[Title/Abstract]) AND ("Unresectable"[Title/Abstract] OR "advanced"[Title/Abstract]) AND ("intrahepatic cholangiocarcinoma"[Title/Abstract] OR "ICC"[Title/Abstract] OR "ICCA"[Title/Abstract])
Web of Science	((TS=((logical) OR (Locoregional) OR (LRT) OR (embolization) OR (Embolization, Therapeutic) OR (Chemoembolization, Therapeutic) OR (chemoembolization) OR (radioembolization) OR (TACE) OR (TARE) OR (hepatic arterial infusion) OR (HAI) OR (intra-arterial therapy) OR (Ablation Techniques) OR (Ablation) OR (MWA) OR (RFA) OR (Cryoablation) OR (Y-90) OR (Radiotherapy) OR (radiation) OR (Chemoradiotherapy) OR (EBRT) OR (SBRT) OR (Chemotherapy) OR (systemic treatment) OR (Systemic Therapy) OR (Sorafenib) OR (Lenvatinib) OR (Atezolizumab and bevacizumab) OR (regorafenib) OR (Cabozantinib) OR (ramucirumab) OR (Nivolumab) OR (Pembrolizumab) OR (PD-1) OR (PD-L1) OR (Combined Modality Therapy))) AND TS=((Unresectable) OR (advanced))) AND TS=((intrahepatic cholangiocarcinoma) OR (ICC) OR (ICCA))
Cochrane Library	#1 (logical):ti,ab,kw OR (Locoregional):ti,ab,kw OR (LRT):ti,ab,kw OR (embolization):ti,ab,kw OR MeSH descriptor: [Embolization, Therapeutic] explode all trees OR (Embolization, Therapeutic):ti,ab,kw OR (chemoembolization):ti,ab,kw OR (radioembolization):ti,ab,kw

	OR (TACE" OR (TARE):ti,ab,kw OR (hepatic arterial infusion):ti,ab,kw OR (HAI):ti,ab,kw OR (intra-arterial therapy):ti,ab,kw OR MeSH descriptor: [Ablation Techniques] explode all trees OR (Ablation Techniques):ti,ab,kw OR (Ablation):ti,ab,kw OR (MWA):ti,ab,kw OR (RFA):ti,ab,kw OR (Cryoablation):ti,ab,kw OR (Y-90):ti,ab,kw OR MeSH descriptor: [Radiotherapy] explode all trees OR (Radiotherapy):ti,ab,kw OR MeSH descriptor: [Radiation] explode all trees OR (radiation):ti,ab,kw OR (EBRT):ti,ab,kw OR (SBRT):ti,ab,kw OR (Chemotherapy):ti,ab,kw OR (systemic treatment):ti,ab,kw OR (Systemic Therapy):ti,ab,kw OR (Sorafenib):ti,ab,kw OR (Lenvatinib):ti,ab,kw OR (Atezolizumab and bevacizumab):ti,ab,kw OR (regorafenib):ti,ab,kw OR (Cabozantinib):ti,ab,kw OR (ramucirumab):ti,ab,kw OR (Nivolumab):ti,ab,kw OR (Pembrolizumab):ti,ab,kw OR (PD-1):ti,ab,kw OR (PD-L1):ti,ab,kw [Word variations have been searched]<br>#2 (Unresectable):ti,ab,kw OR (advanced):ti,ab,kw) [Word variations have been searched]<br>#3 (intrahepatic cholangiocarcinoma):ti,ab,kw OR (ICC):ti,ab,kw OR (ICCA):ti,ab,kw) [Word variations have been searched]<br>#4 #1 AND #2 AND #3
EMBASE	#1 ‘logical’:ti,ab,kw OR ‘Locoregional’:ti,ab,kw OR ‘LRT’:ti,ab,kw OR 'artificial embolization'/exp OR 'artificial embolization':ti,ab,kw OR ‘embolization’:ti,ab,kw OR ‘Embolization, Therapeutic’:ti,ab,kw OR 'chemoembolization'/exp OR ‘chemoembolization’:ti,ab,kw OR 'radioembolization'/exp OR ‘radioembolization’:ti,ab,kw OR ‘TACE" OR ‘TARE’:ti,ab,kw OR ‘hepatic arterial infusion’:ti,ab,kw OR ‘HAI’:ti,ab,kw OR ‘intra-arterial therapy’:ti,ab,kw OR 'ablation therapy'/exp OR 'ablation therapy':ti,ab,kw OR ‘Ablation Techniques’:ti,ab,kw OR ‘Ablation’:ti,ab,kw OR ‘MWA’:ti,ab,kw OR ‘RFA’:ti,ab,kw OR ‘Cryoablation’:ti,ab,kw OR ‘Y-90’:ti,ab,kw OR 'Radiotherapy'/exp OR ‘Radiotherapy’:ti,ab,kw OR 'radiation'/exp OR ‘radiation’:ti,ab,kw OR ‘EBRT’:ti,ab,kw OR ‘SBRT’:ti,ab,kw OR ‘Chemotherapy’:ti,ab,kw OR ‘systemic treatment’:ti,ab,kw OR 'Systemic Therapy'/exp OR ‘Systemic Therapy’:ti,ab,kw OR ‘Sorafenib’:ti,ab,kw OR ‘Lenvatinib’:ti,ab,kw OR ‘Atezolizumab and bevacizumab’:ti,ab,kw OR ‘regorafenib’:ti,ab,kw OR ‘Cabozantinib’:ti,ab,kw OR ‘ramucirumab’:ti,ab,kw OR ‘Nivolumab’:ti,ab,kw OR ‘Pembrolizumab’:ti,ab,kw OR ‘PD-1’:ti,ab,kw OR ‘PD-L1’:ti,ab,kw<br>#2 ‘Unresectable’:ti,ab,kw OR ‘advanced’:ti,ab,kw<br>#3 'intrahepatic cholangiocarcinoma'/exp OR ‘intrahepatic cholangiocarcinoma’:ti,ab,kw OR ‘ICC’:ti,ab,kw OR ‘ICCA’:ti,ab,kw<br>#4 #1 AND #2 AND #3

SUPPLEMENTARY TABLE 2. Newcastle-Ottawa Quality Assessment Scale for cohort studies

Study	Selection				Comparability	Outcome			Total stars	Risk of bias
	Representativeness of the exposed cohort	Selection of the non-exposed cohort	Ascertainment of exposure	Demonstration that outcome of interest was not present at start of study	Comparability of cohorts based on the design or analysis	Assessment of outcome	Was follow-up long enough for outcomes to occur	Adequacy of follow-up of cohorts		
Yang2022	–	–	*	*	**	*	–	*	6	Moderate
Yan2022	–	–	*	*	**	*	–	*	6	Moderate
Sun2021	–	–	*	*	–	*	*	*	5	Moderate
Gairing2021	–	–	*	*	**	*	–	*	6	Moderate
Hu2020	–	–	*	*	**	*	*	*	7	Low
Verma2018	*	*	*	*	**	*	–	*	8	Low
Chang2018	*	*	*	*	**	*	–	*	8	Low
Konstantinidis2016	–	–	*	*	–	*	*	*	5	Moderate
Edeline2015	*	–	*	*	*	*	*	–	6	Moderate
Kim2013	–	–	*	*	–	*	*	*	5	Moderate

*, One star awarded to the study in the item; –, The study was not eligible for a star in the item or did not provide enough information for assessment.

SUPPLEMENTARY TABLE 3. Details of included studies (1).

Study	Title	Journal	Language	Country / Region	Setting	Organization	Study period	Design	Type
Yang2022	Efficacy and Safety of Drug-Eluting Beads Transarterial Chemoembolization Combining Immune Checkpoint Inhibitors in Unresectable Intrahepatic Cholangiocarcinoma: A Propensity Score Matching Analysis	Frontiers in Immunology	English	China	Single center	Sichuan Cancer Hospital, China	May 2019 to August 2021	Retrospective study	Cohort study
Yan2022	Addition of thermal ablation to systemic chemotherapy for the treatment of unresectable intrahepatic cholangiocarcinoma: a propensity score matching analysis	Expert Review of Gastroenterolo gy and Hepatology	English	China	Single center	Department of Minimally Invasive Treatment Center, Fudan University Shanghai Cancer Center	January 2010 to Decem ber 2018	Retrospective study	Cohort study
Sun2021	Efficacy of Transcatheter Arterial Chemoinfusion Combined with Gemcitabine + S-1 Systemic Chemotherapy in Treating Advanced Intrahepatic Cholangiocarcinoma	Minerva Medica	English	China	Single center	Department of Interventional Radiology, Hainan Hospital of PLA General Hospital, Sanya, China	March 2014 to March 2016	Retrospective study	Cohort study

Study	Title	Journal	Language	Country / Region	Setting	Organization	Study period	Design	Type
Gairing2021	The addition of transarterial chemoembolization to palliative chemotherapy extends survival in intrahepatic cholangiocarcinoma	Journal of Clinical Medicine	English	Germany	Single center	Department of Internal Medicine I, University Medical Center of the Johannes Gutenberg University Mainz, 55131 Mainz, Germany	January 2010 to Decem ber 2020	Retrospective study	Cohort study
Hu2020	"Comparison of the efficacy and safety among apatinib plus drug-eluting bead transarterial chemoembolization (TACE), apatinib plus conventional TACE and apatinib alone in advanced intrahepatic cholangiocarcinoma"	American Journal of Translational Research	English	China	Single center	Department of Tumor Interventional Radiology, Fujian Cancer Hospital & Fujian Medical University Cancer Hospital, Fuzhou 350014, China.	Octobe r 2015 to Decem ber 2019	Retrospective study	Cohort study
Verma2018	Chemoradiotherapy versus chemotherapy alone for unresected intrahepatic cholangiocarcinoma: practice patterns and outcomes from the national cancer data	Journal of Gastrointestinal Oncology	English	America	Multicent er	The National Cancer Database (NCDB)	2004-2013	Retrospective study	Cohort study

Study	Title	Journal	Language	Country / Region	Setting	Organization	Study period	Design	Type
	base								
Chang2018	Treatment outcomes for unresectable intrahepatic cholangiocarcinoma: Nationwide, population-based, Cohort study based on propensity score matching with the Mahalanobis metric	Radiotherapy and Oncology	English	China/Taiwan	Multicenter	Taiwan Cancer Registry Database	January 1, 2006 to December 31, 2015	Retrospective study	Cohort study
Konstantinidis2016	Unresectable intrahepatic cholangiocarcinoma: Systemic plus hepatic arterial infusion chemotherapy is associated with longer survival in comparison with systemic chemotherapy alone	Cancer	English	America	Single center	Memorial Sloan Kettering Cancer Center	January 2000 to August 2012	Retrospective study	Cohort study
Edeline2015	Glass Microspheres 90Y Selective Internal Radiation Therapy and Chemotherapy as First-Line Treatment of Intrahepatic Cholangiocarcinoma	Clinical Nuclear Medicine	English	France	Single center	Medical Oncology, Centre Eugène Marquis, Rennes, France	August 2010 to February 2014	Retrospective study	Cohort study
Kim2013	Outcomes of concurrent chemoradiotherapy	Radiation Oncology	English	Korea	Single center	National Cancer Center,	June 2001 to	Retrospective study	Cohort study

Study	Title	Journal	Language	Country / Region	Setting	Organization	Study period	Design	Type
	versus chemotherapy alone for advanced-stage unresectable intrahepatic cholangiocarcinoma					Korea	March 2012		

SUPPLEMENTARY TABLE 3. Details of included studies (2).

Study	Definition of unresectable iCCA	Inclusion criteria	Exclusion criteria	Group	Intervention	Out-comes
Yang2022	Unresectable iCCA included multifocal tumors, extensive regional lymphadenopathy, distant metastases, nonreconstructable vascular involvement, or severe underlying liver parenchymal disease.	1) age between 18 and 80 years; 2) histologically or cytologically confirmed diagnosis of iCCA; 3) ECOG PS of ≤ 2; 4) Child-Pugh class ≤ 7.	1) with other malignancies; 2) previously received TACE, curative resection, ablation, HAIC, other systemic treatment, or radiotherapy; 3) current or previous central nervous system metastasis; 4) mixed feature cHCC-ICC; 5) with current or previous severe cardiovascular disease or coagulation disorders; 6) incomplete clinical or imaging data.	DEB-TACE + ICIs	1. DEB-TACE: 50–80mg doxorubicin-loaded CalliSpheres beads. DEB-TACE was repeated "on demand" in patients with no deteriorating physical status or organ function after contrastenhanced MRI or CT detected viable tumors during follow-up. 2. ICIs (200 mg camrelizumab or sintilimab, every 3 weeks): administration of ICIs was first within one week after the initiated DEB-TACE until severe toxicity, tumor progression, or death.	OS, PFS, ORR, AEs
				Chemotherapy	Intravenous administration comprised cisplatin (25 mg/m^2) followed by gemcitabine (1000 mg/m^2) and performed on day 1 and 8 every 21 days.	
Yan2022	1) newly pathologically diagnosed with iCCA; 2) surgical	1) newly pathologically diagnosed with iCCA; 2) surgical resection was not feasible; 3) liver function of	Patients with a poor physical status and those who underwent pre/posttreatment for lesions were excluded	Ablation-chemotherapy	1. Ablation: radiofrequency ablation (RFA) or microwave ablation (MWA) 2. Chemotherapy:	OS

Study	Definition of unresectable iCCA	Inclusion criteria	Exclusion criteria	Group	Intervention	Out-comes
	resection was not feasible.	Child-Pugh A or B; 4) no prior treatment for iCCA; and 5) Karnofsky Performance Status (KPS) score≥80.			gemcitabine + platinum: n=21, Gemcitabine + S-1: n=4; Gemcitabine + albumin paclitaxel/5-Fluoruracil: n=2; Others: n=9	
				Chemotherapy	Gemcitabine + platinum: n=20, Gemcitabine + S-1: n=2; Gemcitabine + albumin paclitaxel/5-Fluoruracil: n=6; Others: n=8	
Sun2021	NR	NR	NR	TACI + chemotherapy	1. TACI: 5-Fluoruracil + Cisplatin<br>2. Systemic chemotherapy: Gemcitabine + S-1	OS, PFS, ORR, AEs
				Chemotherapy	Gemcitabine + cisplatin + S-1	
Gairing2021	1) patients with primarily resectable and recurrence unresectability; 2) patients with primarily unresectable disease.	NR	NR	TACE + chemotherapy	1. TACE: cTACE (mitomycin C): n=2; DEB-TACE (doxorubicin): n=9; Combination n=3<br>2. Chemotherapy: Gemcitabine: n=1; Gemcitabine + cisplatin/oxaliplatin: n=9; Folinic acid + fluorouracil + oxaliplatin/Capecitabine + oxaliplatin: n=1; Folinic acid + fluorouracil + irinotecan + oxaliplatin: n=1; Other: n=2	OR

Study	Definition of unresectable iCCA	Inclusion criteria	Exclusion criteria	Group	Intervention	Out-comes
				Chemotherapy	Chemotherapy: Gemcitabine: n=7; Gemcitabine + cisplatin/oxaliplatin: n=8; Folinic acid + fluorouracil + oxaliplatin/Capecitabine + oxaliplatin: n=11; Folinic acid + fluorouracil + irinotecan + oxaliplatin: n=1; Other: n=9	
Hu2020	1) pathologically diagnosed with iCCA; 2) TNM stage III~IV according to the American Joint Committee on Cancer (AJCC) 7th Edition Cancer Staging System; 3) confirmed as unresectable disease, or identified as progressed disease after treatment.	1) pathologically diagnosed with iCCA; 2) TNM stage III~IV according to the American Joint Committee on Cancer (AJCC) 7th Edition Cancer Staging System; 3) confirmed as unresectable disease, or identified as progressed disease after treatment; 4) age≥18 years; 5) Eastern Cooperative Oncology Group (ECOG) score≤2; 6) adequate bone marrow function (leukocyte count≥3000 cells/μL, white blood cell ≥3000 cells/mm3, absolute neutrophils ≥1500 cells/μL, platelet count≥50,000 cells/ μL, hemoglobin concentration≥9.0 g/dL); 7)	1) Child-Pugh stage C; 2) heart dysfunction or severe lung dysfunction; 3) active infection; 4) concurrent with pregnancy or lactation (females); 5) allergic to any of the research drugs or reagents.	DEB-TACE + apatinib	1. Oral administration of apatinib at a dose of 500 mg within 1 week before the DEB-TACE operation. 2. DEB-TACE (total doses of gemcitabine and cisplatin were 1 g/m^2 and 65 mg/m^2): ①Gemcitabine and cisplatin infusion②1 g gemcitabine-loaded CalliSpheres beads (containing 0.8 g gemcitabine). If necessary, repeated DEB-TACE treatments were administered. 3. Continued to receive apatinib at a dose of 500 mg after DEB-TACE until	OS, PFS, ORR, AEs

Study	Definition of unresectable iCCA	Inclusion criteria	Exclusion criteria	Group	Intervention	Out-comes
		adequate liver function (total bilirubin≤2 mg/ dL, aspartate aminotransferase and alanine aminotransferase≤5 up to the limit of normal), and patients with biliary tract obstruction should have serum bilirubin levels at <2.0 mg/ dL after treatment by percutaneous hepatic puncture biliary drainage; 8) adequate renal function (creatinine≤2 mg/dL)			disease progression, intolerable adverse effects, or death.	
				cTACE + apatinib	1. Oral administration of apatinib at a dose of 500 mg within 1 week before the DEB-TACE operation. 2. cTACE (total doses of gemcitabine and cisplatin were 1 g/m^2 and 65 mg/m^2): ①Gemcitabine and cisplatin②10 ml lipiodol mixed with 0.8 g gemcitabine. If necessary, repeated cTACE treatments were administered. 3. Continued to receive apatinib at a dose of 500 mg after cTACE until disease progression, intolerable adverse effects, or death.	
				Apatinib	Apatinib at 500 mg daily (orally) until disease progression, intolerable side effects, or death.	
Verma2018	NR	Patients with newly-diagnosed primary iCCA	1) other biliary neoplasms or hepatocellular carcinoma; 2)	Chemoradio therapy	NR	OS

Study	Definition of unresectable iCCA	Inclusion criteria	Exclusion criteria	Group	Intervention	Out-comes
			patients that underwent resection (lobectomy, hepatectomy, wedge/segmental resection, or surgery not otherwise specified); 3) patients with M1 disease, unknown M classification, or in situ disease; 4) patients without known receipt of CT; 5) those with missing RT status; 6) those coded as palliative in the database.	Chemotherapy	NR	
Chang2018	NR	1) an unresectable iCCA diagnosis with a contraindication for surgery; 2) age ≥ 20 years; 3) presence of cholangiocarcinoma; 4) American Joint Committee on Cancer (AJCC) clinical cancer stages I through IV (without metastasis); 5) treatment with an RT dose 4500 ≥ cGy.	1) a history of cancer before iCCA diagnosis; 2) distant metastasis; 3) tumor in unknown intrahepatic or extrahepatic locations; 4) missing sex data; 5)unclear staging; 6) non-CC histology; 7) patients with unresectable iCCA who did not receive sequential CTRT or CCRT after unresectable iCCA diagnosis, did not receivefluoropyrimidine- or gemcitabine-based CT regimens, received RT alone, or underwent therapy for >12 weeks after the diagnosis.	CCRT + chemotherapy	1. RT dose 4500 ≥ cGy 2. Chemotherapy: gemcitabine-based regimens: n=89; fluoropyrimidine-based regimens: n=122	OS
				CTRT + chemotherapy	1. RT dose 4500 ≥ cGy 2. Chemotherapy: gemcitabine-based regimens: n=87; fluoropyrimidine-based regimens: n=124	
				Chemotherapy	Chemotherapy: gemcitabine-based regimens: n=88; fluoropyrimidine-based regimens: n=123	
Konstantinidis2016	"Patients with a histologically	1) patients with a histologically confirmed diagnosis of iCCA	1) patients subjected to resection; 2) patients not treated with	Chemotherapy + HAI	1. HAI agents: floxuridine monotherapy,	OS, ORR

Study	Definition of unresectable iCCA	Inclusion criteria	Exclusion criteria	Group	Intervention	Out-comes
	confirmed diagnosis of iCCA that was not amenable to resection at initial presentation, as determined by attending hepatobiliary surgeons. Unresectable disease included distant metastases, nonreconstructable vascular involvement, or severe underlying liver parenchymal disease."	that was not amenable to resection at initial presentation; 2) having distant metastases, nonreconstructable vascular involvement, or severe underlying liver parenchymal disease.	chemotherapy or treated elsewhere; 3) patients with missing treatment and/or outcome data; 4) prior hepatic radiation or treatment with FUDR; 5)a Karnofsky performance status< 60; 6) first-degree sclerosing cholangitis, Gilbert's disease, portal hypertension, severe hepatic parenchymal dysfunction (encephalopathy, serum albumin < 2.5 g/dL, serum bilirubin ≥1.8 mg/dL, or international normalized ratio >1.5), or portal inflow occlusion; 7) white blood cell count < 3500 cells/mm3; 8) concurrent malignancy (except for localized basal or squamous cell skin cancers); 9) active infection; 10) concurrent pregnancy or lactation (females).		floxuridine/mitomycin, gemcitabine. 2. chemotherapy: gemcitabine regimen, irinotecan regimen, 5-Fluoruracil regimen.	
				Chemotherapy	chemotherapy: gemcitabine regimen, 5-Fluoruracil regimen, others (GX-8951S, platinumbased regimens, and taxol-based regimens).	
Edeline2015	All patients were discussed in a multidisciplinary team meeting specialized in liver malignancies, with liver surgeons, and their disease were judged	1) biopsy-proven iCCA, with no or limited extrahepatic disease, involvement of 50% or less of the liver volume by the tumor; 2) adequate liver function (no cirrhosis or Child-Pugh class A cirrhosis, with bilirubin level ≤35 μmol/L; 3) hepatopulmonary shunt less than 20%; 4) performance status of 2 or lower.		^{90}Y SIRT+ Chemotherapy	1. 90Y SIRT: At the end of the diagnostic angiography, ^{99m}Tcmacroaggregated albumin was injected selectively in the right, left, or segmental hepatic arterial branch to assess the	OS, PFS

Study	Definition of unresectable iCCA	Inclusion criteria	Exclusion criteria	Group	Intervention	Out-comes
	unresectable.				percentage of pulmonary shunting and confirm the absence of digestive uptake. Selective internal radiation therapy was performed 8 to 15 days later during a second angiography, using glass microspheres.<br>2. Chemotherapy: 1) the modified LV5FU2-cisplatin regimen consisted in cisplatin at 50 mg/m^2 on day 1, 5FU bolus at 400 mg/m^2 on day 1, and 5FU continuous infusion at 2400 mg/m^2 upon 46 hours, cycles repeated every 2 weeks, n=4; 2) the GEMOX regimen consisted in gemcitabine 1000 mg/m^2 on day 1 and oxaliplatin 100 mg/m^2 either on day 1 or 2, cycles repeated every 2 weeks, n=13; 3) the gemcitabinecisplatin regimen consisted in cisplatin 25 mg/m^2 onday1and8and	

Study	Definition of unresectable iCCA	Inclusion criteria	Exclusion criteria	Group	Intervention	Out-comes
					gemcitabine 1000 mg/m2 on day 1 and 8, cycles repeated every 3 weeks, n=7.	
	Histopathological or cytologic diagnosis of nonresectable, recurrent, or metastatic iCCA.	1) ABC-02 database iCCA patients corresponding with this study's population; 2) treated in the cisplatin-gemcitabine arm; 3) with first assessment showing stable disease or response	1) patients with metastatic disease; 2)patients with evidence of progression or not evaluated.	Chemotherapy	Cisplatin (25 mg/m^2) followed by gemcitabine (1000 mg/m^2), on days 1 and 8, every 3 weeks for eight cycles.	
Kim2013	Patients were found to have stage IVa (46.7%) or IVb (53.3%) disease according to the seventh edition of the American Joint Committee on Cancer-TNM staging system.	1) patients were found to have stage IVa (46.7%) or IVb (53.3%) disease according to the seventh edition of the American Joint Committee on Cancer-TNM staging system; 2) patients treated with capecitabine + cisplatin	Patients who were treated with other chemotherapy regimens, underwent surgical resection, or received supportive care alone.	CCRT + chemotherapy	1. CCRT was applied in single fractions of 2.0– 3.0 Gy once a day and 5 times a week, with a mean total RT dose of 44.7 Gy (range 25.0–60.0 Gy). Although usual target doses were between 37.5 Gy and 50.0 Gy, several fractions of booster RT were performed in some well-tolerate patients with limit dose of 60.0 Gy.<br>2. Each patient received 1000 mg/m2 oral capecitabine twice daily for the first 14 days of each 21-day cycle, followed by a 7-day rest period, together	OS, PFS, ORR, AEs

Study	Definition of unresectable iCCA	Inclusion criteria	Exclusion criteria	Group	Intervention	Out-comes
					with 30 mg/m2 intravenous cisplatin for 1 hour with standard hydration on days 1 and 8 of each cycle. Patients were continued on chemotherapy until progressive disease or the development of severe toxicity.	
				Chemotherapy	Each patient received 1000 mg/m2 oral capecitabine twice daily for the first 14 days of each 21-day cycle, followed by a 7-day rest period, together with 30 mg/m2 intravenous cisplatin for 1 hour with standard hydration on days 1 and 8 of each cycle. Patients were continued on chemotherapy until progressive disease or the development of severe toxicity.	

SUPPLEMENTARY TABLE 3. Details of included studies (3).

Study	Group	Sample, N	Age, years	Sex (female/male)	Follow-up, months	Child-Pugh class (A/B/C) (n)	ECOG PS(0/1/2/3/4/5) (n)
Yang2022	DEB-TACE + ICIs	20	59 (34–76)**	9/11	7.2 (2.8–28.5)**	16/4/0	8/10/2/0/0/0
	Chemotherapy	20	59 (31–74)**	7/13		19/1/0	5/14/1/0/0/0
Yan2022	Ablation-chemotherapy	36	NR	14/22	NR	NR	NR
	Chemotherapy	36	NR	15/21	NR	NR	NR
Sun2021	TACI + chemotherapy	33	NR	NR	NR	NR	NR
	Chemotherapy	33	NR	NR	NR	NR	NR
Gairing2021	TACE + chemotherapy	14	61.3 (36.7–79.3)**	8/6	18.1 (0.9–107.5)**	NR	13/1/0/0/0/0
	Chemotherapy	59	66.8 (28.8–83.1)**	29/30		NR	NR
Hu2020	DEB-TACE + apatinib	13	55.9±14.3*	7/6	NR	4/9/0	0/9/4/0/0/0
	cTACE + apatinib	12	56.9±9.7*	3/9	NR	4/8/0	0/9/3/0/0/0
	Apatinib	10	58.7±7.8*	2/8	NR	3/7/0	0/7/3/0/0/0
Verma2018	Chemoradiotherapy	666	65 (56–73)***	309/357	10 (0–114)**	NR	NR
	Chemotherapy	2176	65 (56–72)***	1095/1081		NR	NR
Chang2018	CCRT + chemotherapy	211	60.11±10.20	81/130	10.27 (9.37)***	NR	NR
	CTRT + chemotherapy	211	60.13±10.59	80/131	8 (4.97)***	NR	NR
	Chemotherapy	211	60.80±10.67	84/127	7.1 (5.8)***	NR	NR
Konstantinidis2016	Chemotherapy + HAI	78	62 (30–84)**	47/31	NR	NR	NR
	Chemotherapy	26	62 (30–84)**	13/13	NR	NR	NR
Edeline2015	^{90}Y SIRT+ Chemotherapy	24	64 (29–79)**	10/14	19.0 (NR)**	NR	12/9/3/0/0/0
	Chemotherapy	33	NR	NR	15.7 (NR)**	NR	NR
Kim2013	Chemotherapy	25	56 (32–75)**	6/19	NR	NR	10/14/1/0/0
	DEB-TACE + ICIs	67	58 (26–78)**	14/53	NR	NR	39/24/4/0/0

Supplementary Table 3. Details of included studies (4).

Study	Group	OS			PFS		
		**Definition**	**HR (95% CI)**	**Median (95% CI), months**	**Definition**	**HR (95% CI)**	**Median (95% CI), months**
Yang2022	DEB-TACE + ICIs	From inpatients to death or the last follow-up.	0.347 (0.139–0.867)	13.2 (4.977–21.423)	From inpatients to disease progression or death.	0.025 (0.005–0.116)	17.0(9.6–24.4)
	Chemotherapy		Reference	7.6 (6.317–8.883)		Reference	4.5(3.2–5.8)
Yan2022	Ablation-chemotherapy	From the diagnosis of iCCA to death or the last follow-up.	0.531 (0.312–0.904)	15.233 (11.722–18.745)	NR		
	Chemotherapy		Reference	7.967 (2.479–13.455)			
Sun2021	TACI + chemotherapy	NR	0.51 (0.31–0.82)	NR	NR	0.59 (0.36–0.96)	NR
	Chemotherapy		Reference	NR		Reference	NR
Gairing2021	TACE + chemotherapy	From diagnosis of unresectability to death or the last follow-up.	0.41 (0.22–0.79)	26.2 (NR)	NR		
	Chemotherapy		Reference	13.1 (NR)			
Hu2020	DEB-TACE + apatinib	From the initiation of the study treatment to death.	0.005 (0.001–0.043)	19.3 (12.6–26.0)	From initiation of study treatment to disease progression or death.	0.025 (0.005–0.116)	17.0 (9.6–24.4)
	cTACE + apatinib		0.013 (0.002–0.089)	14.0 (10.2–17.8)		0.090 (0.025–0.325)	10.3 (6.7–13.9)
	Apatinib		Reference	6.5(4.7–8.3)		Reference	4.5 (3.2–5.8)
Verma2018	Chemoradiotherapy	From the diagnosis of iCCA to death or the last follow-up.	0.72 (0.65–0.81)	13.6 (12.3–15.7)	NR		
	Chemotherapy		Reference	10.5 (10.0–11.5)			
Chang2018	CCRT + chemotherapy	NR	0.65 (0.59–0.71)	NR	NR		

Study	Group	OS			PFS		
		Definition	HR (95% CI)	Median (95% CI), months	Definition	HR (95% CI)	Median (95% CI), months
	CTRT + chemotherapy		0.95 (0.83–1.48)	NR			
	Chemotherapy		Reference	NR			
Konstantinidis2016	Chemotherapy + HAI	From the diagnosis of iCCA to death or the last follow-up.	0.36 (0.22–0.59)	30.8 (NR)	NR		
	Chemotherapy		Reference	18.4 (NR)			
Edeline2015	90Y SIRT+ Chemotherapy	From the initiation of the study treatment to death.	0.42 (0.19–0.92)	NR	From initiation of study treatment to disease progression or death.	0.70 (0.38–1.29)	16.0 (12.1–20.0)
	Chemotherapy		Reference	NR		Reference	11.3 (8.6–14.0)
Kim2013	Chemotherapy	From the diagnosis of iCCA to death or the last follow-up.	0.63 (0.40–1.00)	9.3 (7.6–11.0)	From initiation of study treatment to disease progression or death.	0.46 (0.29–0.73)	4.3 (3.3–5.4)
	DEB-TACE + ICIs		Reference	6.2 (4.1–8.2)		Reference	1.9 (1.3–2.4)

SUPPLEMENTARY TABLE 3. Details of included studies (5).

Study	Group	ORR		AEs	
		Evaluation criteria	Events, n (%)	Evaluation criteria	Categories
Yang2022	DEB-TACE + ICIs<br>Chemotherapy	Modified Response Evaluation Criteria in Solid Tumors (mRECIST)	11 (55.0)<br>4 (20.0)	National Cancer Institute Common Toxicity Criteria for Adverse Events (NCI-CTCAE) Version 5.0	Leukopenia, Neutropenia, Reduced hemoglobin level, Thrombocytopenia, Increased AST, Increased ALT, Hyperbilirubinemia, Hypoalbuminemia, Nausea, Vomiting, Anorexia, Fatigue, Constipation, Abdominal pain, Alopecia, Rash, Hypothyroidism, Reactive cutaneous capillary endothelial proliferation
Yan2022	Ablation-chemotherapy<br>Chemotherapy	NR			
Sun2021	TACI + chemotherapy<br>Chemotherapy	NR	12 (36.4)<br>9 (27.3)	NR	Erythra, Neutropenia, Thrombocytopenia, Anemia, Nausea and vomiting, Diarrhea, Liver dysfunction, Oral mucositis
Gairing2021	TACE + chemotherapy<br>Chemotherapy	NR			
Hu2020	DEB-TACE + apatinib<br>cTACE + apatinib<br>Apatinib	Response Evaluation Criteria in Solid Tumours (RECIST) 1.1	11 (84.6)<br>9 (75.0)<br>4 (40.0)	NCI-CTCAE Version 4.03	Fatigue, Anorexia, Diarrhea, Hoarseness, Hypertension, Hand-Foot Syndrome, Mucositis, Proteinuria, Hypoproteinemia, Hyperbilirubinemia, ALT increase, Thrombocytopenia, Vomiting, AST increase, Anemia, Neutropenia
Verma2018	Chemoradiotherapy<br>Chemotherapy	NR			
Chang2018	CCRT + chemotherapy<br>CTRT + chemotherapy<br>Chemotherapy	NR			

Study	Group	ORR		AEs	
		Evaluation criteria	Events, n (%)	Evaluation criteria	Categories
Konstantinidis2016	Chemotherapy + HAI<br>Chemotherapy	NR	47 (59)<br>7 (39)	NR	
Edeline2015	90Y SIRT+ Chemotherapy<br>Chemotherapy	NR			
Kim2013	Chemotherapy<br>DEB-TACE + ICIs	RECIST 1.0	1 (4.0)<br>3 (4.6)	NCI-CTCAE Version 3.0	Neutropenia, Thrombocytopenia, Anemia, Anorexia, Nausea, Vomiting, Asthenia, Dyspnea, Peripheral neuropathy, Hand-foot syndrome

95% CI = 95% confidence intervals; AEs = Adverse events; ALT = Alanine transaminase; AST = Aspartate aminotransferase; cHCC-CCA = Combined hepatocellular carcinoma - cholangiocarcinoma; CCRT = Concurrent chemoradiation therapy; cTACE = Conventional transarterial chemoembolization; DEB-TACE = Transarterial chemoembolization with drug-eluting beads; EBRT, external beam radiation therapy; ECOG PS = Eastern Cooperative Oncology Group performance score; HAIC = Hepatic arterial infusion chemotherapy; HAI = Hepatic arterial infusion; HR = hazard ratio; iCCA = Intrahepatic cholangiocarcinoma; ICIs = Immune checkpoint inhibitors; MWA = Microwave ablation; NR = Not reported; NCI-CTCAE = National Cancer Institute Common Toxicity Criteria for Adverse Events; OS = Overall survival; ORR = Objective response rate; PFS = Progression-free survival; RECIST = Response Evaluation Criteria in Solid Tumors; RFA = Radiofrequency ablation; SYS = Systemic chemotherapy; TACE = Transarterial chemoembolization; TACI = Transarterial chemoinfusion; ^{90}Y SIRT = Yttrium-90 selective internal radiotherapy; *, Mean ± SD; **, Median (range); ***, median (interquartile range)

SUPPLEMENTARY TABLE 4. Summary of adverse events following therapies.

		LRT + ST				ST				
**Adverse events**	**Study**	**None, n (%)**	**Grade I-II, n (%)**	**Grade III-IV, n (%)**	**Total, N**	**None**	**Grade I-II, n (%)**	**Grade III-IV, n (%)**	**Total, N**	**P-value**
Neutropenia	Yang2022	19 (95.0)	1 (5.0)	0 (0.0)	20	13 (65.0)	6 (30.0)	1 (5.0)	20	0.018[&]
	Sun2021	11 (33.3)	16 (48.5)	6 (18.2)	33	12 (36.4)	14 (42.4)	7 (21.2)	33	NR
	Hu2020	6 (46.2)	6 (46.2)	1 (7.7)	13	8 (80.0)	1 (10.0)	1 (10.0)	10	NR
	Kim2013	13 (52.0)	12 (48.0)		25	61 (91.0)	6 (9.0)		67	0.001[&]
	Sum	49 (53.8)	42 (46.2)		91	94 (72.3)	36 (27.7)		130	0.533[$]
Thrombocytopenia	Yang2022	18 (90.0)	2 (10.0)	0 (0.0)	20	14 (70.0)	5 (25.0)	1 (5.0)	20	0.114[&]
	Sun2021	27 (81.8)	5 (15.2)	1 (3.0)	33	25 (75.8)	7 (21.2)	1 (3.0)	33	NR
	Hu2020	6 (46.2)	4 (30.8)	3 (23.1)	13	7 (70.0)	1 (10.0)	2 (20.0)	10	NR
	Kim2013	8 (32.0)	17 (68.0)		25	31 (46.3)	36 (53.7)		67	0.218[&]
	Sum	59 (64.8)	32 (35.2)		91	77 (59.2)	53 (40.8)		130	0.925[$]
Anemia	Yang2022	19 (95.0)	1 (5.0)	0 (0.0)	20	17 (85.0)	2 (10.0)	1 (5.0)	20	0.292[&]
	Sun2021	19 (57.6)	12 (36.4)	2 (6.1)	33	20 (60.6)	10 (30.3)	3 (9.1)	33	NR
	Hu2020	2 (15.4)	11 (84.6)	0 (0.0)	13	7 (70.0)	3 (30.0)	0 (0.0)	10	NR
	Kim2013	12 (48.0)	13 (52.0)		25	42 (62.7)	25 (37.3)		67	0.203[&]
	Sum	52 (57.1)	39 (42.9)		91	86 (66.2)	44 (33.8)		130	0.322[$]
Anorexia	Yang2022	18 (90.0)	2 (10.0)	0 (0.0)	20	16 (80.0)	4 (20.0)	0 (0.0)	20	0.376[&]
	Hu2020	8 (61.5)	5 (38.5)	0 (0.0)	13	7 (70.0)	3 (30.0)	0 (0.0)	10	NR
	Kim2013	9 (36.0)	16 (64.0)		25	39 (58.2)	28 (41.8)		67	0.058[&]
	Sum	35 (60.3)	23 (39.7)		58	62 (63.9)	35 (36.1)		97	0.192[$]
Vomiting	Yang2022	12 (60.0)	6 (30.0)	2 (10.0)	20	11 (55.0)	7 (35.0)	2 (10.0)	20	0.749[&]
	Hu2020	6 (46.2)	6 (46.2)	1 (7.7)	13	9 (90.0)	1 (10.0)	0 (0.0)	10	NR
	Kim2013	22 (88.0)	3 (12.0)		25	62 (92.5)	5 (7.5)		67	0.678[&]
	Sum	40 (69.0)	18 (31.0)		58	82 (84.5)	15 (15.5)		97	0.133[$]
Nausea vomiting	Sun2021	24 (72.7)	8 (24.2)	1 (3.0)	33	20 (60.6)	11 (33.3)	2 (6.1)	33	NR
	Hu2020	6 (46.2)	6 (46.2)	1 (7.7)	13	9 (90.0)	1 (10.0)	0 (0.0)	10	NR
	Sum	30 (65.2)	16 (34.8)		46	29 (67.4)	14 (32.6)		43	NR
Diarrhea	Sun2021	29 (87.9)	4 (12.1)	0 (0.0)	33	26 (78.8)	7 (21.2)	0 (0.0)	33	NR

Adverse events	Study	LRT + ST None, n (%)	LRT + ST Grade I-II, n (%)	LRT + ST Grade III-IV, n (%)	LRT + ST Total, N	ST None	ST Grade I-II, n (%)	ST Grade III-IV, n (%)	ST Total, N	P-value
	Hu2020	11 (84.6)	2 (15.4)	0 (0.0)	13	9 (90.0)	1 (10.0)	0 (0.0)	10	NR
	Sum	40 (87.0)	6 (13.0)	0 (0.0)	46	35 (81.4)	8 (18.6)	0 (0.0)	43	NR
Hand-foot syndrome	Hu2020	3 (23.1)	7 (53.8)	3 (23.1)	13	2 (20.0)	6 (60.0)	2 (20.0)	10	NR
	Kim2013	19 (76.0)	6 (24.0)		25	64 (95.5)	3 (4.5)		67	0.011&
	Sum	22 (57.9)	16 (42.1)		38	66 (85.7)	11 (14.3)		77	NR
Rash	Yang2022	17 (85.0)	3 (15.0)	0 (0.0)	20	19 (95.0)	1 (5.0)	0 (0.0)	20	0.292&
	Sun2021	25 (75.8)	8 (24.4)	0 (0.0)	33	27 (81.8)	6 (18.2)	0 (0.0)	33	NR
	Sum	42 (79.2)	11 (20.8)	0 (0.0)	53	46 (86.8)	7 (13.2)	0 (0.0)	53	NR
Fatigue	Yang2022	12 (60.0)	5 (25.0)	3 (15.0)	20	13 (65.0)	4 (20.0)	3 (15.0)	20	0.744&
	Hu2020	5 (38.5)	7 (53.8)	1 (7.7)	13	4 (40.0)	5 (50.0)	1 (10.0)	10	NR
	Sum	17 (51.5)	12 (36.4)	4 (12.1)	33	17 (56.7)	9 (30.0)	4 (13.3)	30	NR
Hypoproteinemia	Yang2022	15 (75.0)	5 (25.0)	0 (0.0)	20	16 (80.0)	4 (20.0)	0 (0.0)	20	0.705&
	Hu2020	0 (0.0)	13 (100)	0 (0.0)	13	2 (20.0)	8 (80.0)	0 (0.0)	10	NR
	Sum	15 (45.5)	18 (54.5)	0 (0.0)	33	18 (60.0)	12 (40.0)	0 (0.0)	30	NR
Hyperbilirubinemia	Yang2022	14 (70.0)	6 (30.0)	0 (0.0)	20	16 (80.0)	4 (20.0)	0 (0.0)	20	0.465&
	Hu2020	3 (23.1)	7 (53.8)	3 (23.1)	13	5 (50.0)	3 (30.0)	2 (20.0)	10	NR
	Sum	17 (51.5)	13 (39.4)	3 (9.1)	33	21 (70.0)	7 (23.3)	2 (6.7)	30	NR
ALT increased	Yang2022	11 (55.0)	5 (25.0)	4 (20.0)	20	15 (75.0)	3 (15.0)	2 (10.0)	20	0.185&
	Hu2020	1 (7.7)	8 (61.5)	4 (30.8)	13	3 (30.0)	6 (60.0)	1 (10.0)	10	NR
	Sum	12 (36.4)	13 (39.4)	8 (24.2)	33	18 (60.0)	9 (30.0)	3 (10.0)	30	NR
AST increased	Yang2022	11 (55.0)	6 (30.0)	3 (15.0)	20	15 (75.0)	3 (15.0)	2 (10.0)	20	0.185&
	Hu2020	0 (0.0)	8 (61.5)	5 (38.5)	13	3 (30.0)	6 (60.0)	1 (10.0)	10	NR
	Sum	11 (33.3)	14 (42.4)	8 (24.2)	33	18 (60.0)	9 (30.0)	3 (10.0)	30	NR
Nausea	Yang2022	14 (70.0)	6 (30.0)	0 (0.0)	20	12 (60.0)	8 (40.0)	0 (0.0)	20	0.507&
	Kim2013	17 (68.0)	8 (32.0)		25	51 (76.1)	16 (23.9)		67	0.430&
	Sum	31 (68.9)	14 (31.1)		45	63 (72.4)	24 (27.6)		87	NR
Liver dysfunction	Sun2021	15 (45.5)	15 (45.5)	3 (9.1)	33	20 (60.6)	12 (36.4)	1 (3.0)	33	NR
Oral mucositis	Sun2021	17 (51.5)	11 (33.3)	5 (15.2)	33	17 (51.5)	13 (39.4)	3 (9.1)	33	NR

Adverse events	Study	LRT + ST None, n (%)	LRT + ST Grade I-II, n (%)	LRT + ST Grade III-IV, n (%)	LRT + ST Total, N	ST None	ST Grade I-II, n (%)	ST Grade III-IV, n (%)	ST Total, N	P-value
Hoarseness	Hu2020	10 (76.9)	3 (23.1)	0 (0.0)	13	7 (70.0)	3 (30.0)	0 (0.0)	10	NR
Hypertension	Hu2020	1 (7.7)	8 (61.5)	4 (30.8)	13	2 (20.0)	6 (60.0)	2 (20.0)	10	NR
Mucositis	Hu2020	10 (76.9)	3 (23.1)	0 (0.0)	13	6 (60.0)	4 (40.0.)	0 (0.0)	10	NR
Proteinuria	Hu2020	1 (7.7)	12 (92.3)	0 (0.0)	13	4 (40.0)	5 (50.0)	1 (10.0)	10	NR
Asthenia	Kim2013	19 (76.0)	6 (24.0)		25	39 (58.2)	28 (41.8)		67	0.116&
Dyspnea	Kim2013	8 (32.0)	17 (68.0)		25	31 (46.3)	36 (53.7)		67	0.218&
Peripheral neuropathy	Kim2013	22 (88.0)	3 (12.0)		25	64 (95.5)	3 (4.5)		67	0.339&
Leukopenia	Yang2022	19 (95.0)	1 (5.0)	0 (0.0)	20	12 (60.0)	6 (30.0)	2 (10.0)	20	0.028&
Constipation	Yang2022	18 (90.0)	2 (10.0)	0 (0.0)	20	19 (95.0)	1 (5.0)	0 (0.0)	20	0.548&
Abdominal pain	Yang2022	14 (70.0)	4 (20.0)	2 (10.0)	20	17 (85.0)	3 (15.0)	0 (0.0)	20	0.256&
Alopecia	Yang2022	18 (90.0)	2 (10.0)	0 (0.0)	20	17 (85.0)	2 (10.0)	1 (5.0)	20	0.633&
Hypothyroidism	Yang2022	15 (75.0)	5 (25.0)	0 (0.0)	20	20 (100.0)	0 (0.0)	0 (0.0)	20	0.017&
RCCEP	Yang2022	15 (75.0)	5 (25.0)	0 (0.0)	20	20 (100.0)	0 (0.0)	0 (0.0)	20	0.017&

ALT = Alanine transaminase; AST = Aspartate aminotransferase; LRT + ST = locoregional therapy combined with systemic therapy; NR, Not reported; RECPP = Reactive cutaneous capillary endothelial proliferation; ST = Systemic therapy; &, *p-value* extracted from the original article; $, *p-value* calculated from this meta-analysis.
